# Perspectives on male partner notification and treatment for syphilis among antenatal women and their partners in Kampala and Wakiso districts, Uganda

**DOI:** 10.1186/s12879-019-3695-y

**Published:** 2019-02-06

**Authors:** Edith Nakku-Joloba, Juliet Kiguli, Christine Nalwadda Kayemba, Adeline Twimukye, Joshua Kimeze Mbazira, Rosalind Parkes-Ratanshi, Monica Birungi, Joshua Kyenkya, Josaphat Byamugisha, Charlotte Gaydos, Yukari C. Manabe

**Affiliations:** 1 School of Public Health, Makerere University College of Health Sciences, Kampala, Uganda; 2 STD Clinic/Ward 12, Mulago Hospital, Kampala, Uganda; 3 Infectious Diseases Institute, Makerere University College of Health Sciences, Kampala, Uganda; 4 Institute of Public Health, University of Cambridge, Forvie Site, Cambridge, CB2 0SR UK; 5 School of Medicine, Makerere University College of Health Sciences, Kampala, Uganda; 6 Department of Medicine, Division of Infectious Diseases, Johns Hopkins University, Baltimore, MD USA

**Keywords:** Maternal syphilis, Partner notification, Socio-cultural factors, Resource constrained settings

## Abstract

**Background:**

Syphilis screening can be successfully integrated into antenatal clinics, and potentially avert significant morbidity and mortality to unborn infants. A minority of male partners report for testing and treatment, increasing the likelihood of reinfection. We conducted a qualitative study to understand factors influencing male partners to seek treatment after syphilis notification by their pregnant partners.

**Methods:**

A purposeful sample of 54 adults who participated in the STOP (Syphilis Treatment of Partners) study was stratified by gender (24 women, 30 male partners) and enrolled for in-depth interviews which were audio recorded, transcribed, and analyzed using the thematic approach.

**Results:**

The participants’ median age (IQR) was 32 years (25–44), 87% were married, and 57.4% (31/74) had attained secondary education. Fourteen of 22 (63%) female participants reported that they sometimes experienced domestic violence. Male participant’s knowledge of syphilis and their perception of their valued role as responsible fathers of an unborn baby facilitated return. Female’s fear of partner‘s violence and poor communication between partners, were barriers against delivery of the notification forms to partners and subsequent treatment of partners. For men, fear of injection pain, perceptions of syphilis as a genetic disease and as a woman’s problem, busy work schedules, poor access to good STD services, shared facilities with women in clinics, as well as HIV-related stigma were important barrier factors.

**Conclusions:**

The return to the clinic for treatment of male partners after partner notification by infected pregnant women, was low due to limited knowledge about syphilis, fear of painful injection, fears of domestic violence, lack of communication skills (individual characteristics) and syphilis disease characteristics such as signs and symptoms. This, combined with health services characteristics such as structural barriers that hinder male partner treatment, low access, low capacity, work/time challenges, inadequate laboratory services and low clinic personnel capacity; threatens efforts to eliminate mother-to-child infection of syphilis. Improved public messaging about syphilis, better services, legal and policy frameworks supporting STD notification and treatment in resource-constrained settings are needed for effective STD control.

**Trial registration:**

Clinicaltrials.gov NCT02262390., Date Registered October 8 2014.

## Background

Syphilis is a cause of preventable morbidity and mortality in infants in Sub-Saharan Africa (SSA) where 2.5–17% of pregnant women are infected with syphilis [1, 2]. It is estimated that 53–82% of women with untreated syphilis have adverse birth outcomes compared to only 10–21% among uninfected women [3]. Untreated partners of women are a source of reinfection. Increasing partner uptake of syphilis screening and treatment should decrease the risk of re-infection and protect subsequent pregnancies from syphilis re-infection [4]. Uganda’s Ministry of Health (MoH) has a partner notification program as part of the management of STD and syphilis. Free partner notification and treatment is provided with provision of a numbered notification slip. An earlier study showed excellent uptake of syphilis screening, but a minority of partners came in for testing and treatment [5].

The “Comparison of Different Partner Notification Strategies after Antenatal Syphilis Screening” also known as “Syphilis Treatment of Partners (STOP) Trial” was conducted from August 2014 to November 2015 at the Infectious Diseases Institute (IDI), Makerere University. In the STOP trial after one year of study, only 18% of male partners returned for the treatment which was not different by three study arms, where participants received either a paper slip to give to their partner, paper slip plus weekly SMS reminders, or paper slip plus weekly phone calls. We conducted a qualitative study to understand the socio-cultural factors that influenced the male partner compliance with syphilis testing and treatment.

In the pathway model framework detailed by Axel Kroege [6], and guided by the World Health Organization for studies on STD management (https://www.who.int/hiv/topics/en/HealthcareSeeking.pdf), investigators examine or investigate pathway processes to the desired health seeking behavior and the factors that are barriers to or support these processes. The general areas of; i) characteristics of the subject ii) characteristics of the disease, and iii) characteristics of the health service give the overarching areas under which factors considered as barriers to or facilitators to the use of the health seeking behavior of interest are examined. In this study the health behavior of interest is male partners’ return to clinics after notification and receiving syphilis treatment after partner notification. We adapted the framework to the local context supported by a literature review in the area as well as based on WHO guidelines regarding protocols in STD research that seek to understand the behavior of health seeking of STD care.

## Methods

We conducted an exploratory study among women participants enrolled in the STOP trial and their male partners who consented to participate from May to November 2016. The study involved persons enrolled from three study sites in the STOP study; the Infectious Disease Institute (IDI) Sexual and Reproductive Health (SRH) Clinic, Mulago National Referral Hospital; Antenatal and the STD Clinics in Kampala and the Kasangati Health Center IV Antenatal Clinic in Wakiso District. We enrolled women and men aged 18 years and above from the STOP Trial participant register who consented to the study. In the design of the study, we used parts of the model described by Axel Kroeger, which we tried to adapt to the local context and literature review in the area as well as on the WHO guidelines regarding protocols in STD research that seek to understand the health seeking behavior of people for STD care.

### Study participants and selection

A list of eligible participants was generated (women and men) from the main “STOP study” database of 430 individuals who had been provided with syphilis treatment. We then purposefully selected 24 women who received syphilis treatment (based on whether their partners had returned or not) enrolled from the three study sites making sure all sites were represented. We included 12 whose partners had returned for treatment and 12 whose partners had not returned for treatment. In addition, 30 men who had been earlier invited to receive syphilis treatment under the main STOP trial were selected from the study database to include 15 men who were notified and who did return and 15 men who did not attend clinic for syphilis treatment. Participants’ telephone contacts were retrieved and through telephone calls, both men and women were invited for study interviews at the IDI clinic at Mulago. Interviews were scheduled at a time convenient to participants.

### Data collection

We collected data using in-depth interviews with prepared interview guides. All interviews were conducted in Luganda language. Researchers explored individual’s experience of syphilis as a disease, knowledge, management of the disease and attitudes towards partner notification for syphilis treatment. We also explored reasons as to why male participants came or did not come to the clinic after partner notification. Prior to data collection, staff were trained on the protocol and study tools were translated into Luganda, the main language spoken in the catchment area of the study clinics. Study tools were pre-tested on two men and two women and adjusted by rearranging the questions to maintain a good flow of the interviews. Each participant was assigned an identification code which was subsequently used on transcripts.

### Data analysis and interpretation

All 54 audio interviews were transcribed verbatim and typed into Microsoft Word after translation. The research team read through the typed transcripts several times and filled in gaps by listening back to the audio recordings. Team members read the interview transcripts and data was coded into meaning units. Major themes and sub-themes were developed using a master sheet. The data were analyzed and interpreted using the thematic approach.

Interviews were conducted by 2 research assistants and 1 senior scientist and supervisor. Four of the research team members read all the transcripts and identified general themes and categories. We used a bottom-up approach to identify the themes and categories. Transcripts were read and coded manually. We then developed an explicit summary describing each category and theme (Additional file 1) The framework for analysis was developed based on a preliminary analysis of transcripts, was informed by the research questions as well as by information from persons working in the STD clinic regarding possible reasons for male partners not returning after notification.

In the next step we sorted quotes from the transcripts, based on their thematic similarities. We then examined the degree to which these themes were distributed across gender. Quotations and key phrases are highlighted in the findings. Descriptive statistical analysis was performed on the socio-demographic data in Excel.

### Validity and reliability

To maximize validity, we employed expert medical and social scientists with experience in qualitative research and fluent in English and Luganda. Participants were given opportunity to express themselves, in confidence, at length and freely, regarding study questions. Two trained social scientists (AT and AMB) as research assistants supported ENJ, JK and CNK in the data collection. A standardized interview guide was developed and used. Translation of the guide was done by a professional translator, reviewed by the investigators and then finalized. The two interviewers were fluent Luganda and English speakers. Reliability was ensured by use of standardized study tools, audio recording of the interviews, transcription and re-reading of the transcripts.

### Ethical issues

Ethical approval was obtained from the Joint Clinical Research Centre Institutional Review Board (IRB) and the Uganda National Council for Science and Technology (HS1681), Johns Hopkins IRB (NA_00012998 / CR00015330) and was registered at clinicaltrials.gov (NCT02262390). All study participants gave written informed consent and received reimbursement.

## Results

### Socio-demographic characteristics

Median age was 32 (IQR 25–44) years. Most participants were self-employed 26/54 (48%) and married 47/54(87%). Majority of participants had attained secondary education 31/54 (57%) and 14/54 (26%) had a primary eucation. Twenty-two of the 54 participants (41%) reported that they sometimes experienced domestic violence. Of these, 14 women out of the 22 (two had missing data on domestic violence), reported experiencing domestic violence.

All men who attended clinic after partner notification were given syphilis treatment after the interview. Table 1 shows characteristics of study participants.

**Table 1:** Socio-demographic characteristics of study participants in the STOP qualitative study

Characteristic	Frequency N=54	Percentage
Male	30	55.5
Female	24	44.4
Age		
20-24	8	14.8
25-29	10	18.5
30-34	18	33.3
35-39	6	11.1
40-44	9	16.7
45-49	1	1.9
50+	2	3.7
Occupation		
Unemployed	8	14.8
Self employed	26	48.1
Employed	17	31.5
Casual Laborer	2	3.7
Missing	1	1.9
Marital status		
Single	4	7.4
Married	47	87.0
Separated/Divorced	2	3.7
Widow/widower	1	1.9
Period of marriage in years		
0-5	19	35.2
6-10	14	25.9
11-15	9	16.7
16+	5	9.3
Missing	7	13.0
Woman staying with partner		
Yes	44	81.5
No	9	16.7
Missing data	1	1.9
Number of children		
None	3	5.6
1-3	29	53.7
4-6	17	31.5
7+	4	7.4
Missing	1	1.9
Education		
None	3	5.6
Primary	14	25.9
Secondary	31	57.4
Tertiary	6	11.1
Religion		
Anglican Christian	11	20.4
Catholic Christian	21	38.9
Muslim	12	22.2
Born Again Christian	10	18.5
History of domestic violence		
No	25	46.3
Sometimes	22	40.7
Missing	7	13.0
Temper of woman’s partner		
Controllable	26	48.1
High Tempered	28	51.9

### Perceptions and knowledge of syphilis and treatment for syphilis

Knowledge of and perceptions about syphilis as a disease affected men’s reporting after notification. Many participants understood syphilis was a sexually transmitted disease with signs and symptoms;

*“Syphilis can be got by the married ones and those ones who are not married because it is contracted through having sex with an infected person.”* (Female, in their 30’s, partner did not return, accessed ANC at Site 1).

*When you have syphilis, you get a smelly discharge, itches in the private parts as well as genital sores.* (Male, in their 20’s, did not return, partner accessed ANC at Site 1).

Men reported fear of finding out HIV status as a reason for not returning after partner notification, thinking they would be tested for HIV, yet they were not ready to be tested.

“*Syphilis is associated with HIV/AIDS that if someone has syphilis it’s obvious you must have HIV/AIDS. Some fear that they may be stigmatized that since they have syphilis, it’s obvious that they have HIV/AIDS. Some are very polygamous so fear to be asked about all their partners.”* (*,* Male, in their 40’s, did not return, partner accessed ANC at Site 1).

*“Most men think that when they come, they will be tested for HIV yet majority do not want to know their HIV status.”* (Male, in their 30’s, did not return, partner accessed ANC at Site 1).

Women similarly reported that their partners were afraid of being diagnosed as HIV-positive when asked to go to the doctor. *“You see those men each time you tell them that the doctor needs you, he only thinks of one thing, HIV! He says, they are calling me? It is ‘slim’ (HIV), so convincing him is a problem.”* (Female, in their 20’s, partner did not return, accessed care at Site 2).

Some participants reported a belief that syphilis is a familial (genetic) disease rather than sexually transmitted so there was no need to go for treatment, *“He told me he fears the hospital and is aware that all in their family, and have syphilis. It’s a family disease, so (there is) no need to go for treatment.”* (Female, in their 20’s, partner did not return, accessed care at Site 1).

*“She had told me previously that syphilis was common in their family, that at their home, her father had it, some of her sisters have miscarried because of it”.* (Male, in their 40’s, returned, partner accessed ANC at Site 1).

Syphilis infection was described by the unique characteristic of the medicine used to treat it; some ascribed their partners ‘not-returning’ to fear of the painful injection for its treatment. Participants described; *“I am sure it is that painful injection that scares men and makes them not to come”.*" *.* (Male, in their 40’s, returned, partner accessed ANC at Site 1).

*“I also think he did not come because of that injection, he fears it a lot because it’s very painful.”* (Female, in their 30’s, partner did not return, accessed ANC at Site 1).

Some participants thought that treating the male partners for syphilis is not necessary if the woman had received syphilis treatment. A male participant said;

*“When your wife is treated there is no need for the man to undergo treatment.” (Male, 28 years, Did not Come). “She told me that she was diagnosed with syphilis, she didn’t give me so much information about syphilis, she was always in the village and I was very busy working. She didn’t give me any document and since I didn’t have anything paining me, I didn’t come for treatment.”* (Male, in their 20’s, did not return, partner accessed ANC at Site 1) .

However, other men recognized the danger of non-treatment and came to the clinic after notification. One male participant explained, “*You know us men, most times we are big-headed but if they tested her and she was treated yet I was not, however much they treat her it’s a waste of time.”* (Male, in their 30’s, returned, partner accessed ANC at Site 1).

Participants reported they believed syphilis treatment was beneficial and led to positive outcomes. Many believed receiving treatment is a sign of taking responsibility for one’s health. They frequently mentioned ‘feeling good and comfortable’, ‘no more pain’, ‘giving birth to healthy babies free from syphilis’, ‘improved quality of life’ and ‘reduced opportunistic infections after syphilis treatment’ as benefits of syphilis treatment. They said that syphilis treatment “strengthened couple’s love” and saved their finances; as a man who came after notification said;

*“We prevent infection in our family and save our money. When I have a health problem I go to hospital, it means my income has reduced. That is the key issue why I say treatment is essential.”* (Male, in their 30’s, returned, partner accessed care at Site 2).

Another mentioned*, “When I received treatment for syphilis the fevers ceased. My bones stopped cracking. So when cured, your health improves so that even when you get other illnesses, they do not affect you so badly.”*(Male, in their 30’s, returned, partner accessed ANC at Site 1).

Some men who returned after notification were motivated by the knowledge that treatment could prevent transmission to unborn children and commitment to their wives and children.

“*The good (thing) about treating syphilis is that if syphilis is tested and treated then a woman can give birth to a healthy babies. Those kids who are born with wounds, it is because their parents had syphilis and they refused to treat it*” (Male, in their 40’s, returned, Partner accessed ANC at Site 3).

*“I told him that the doctors said they would not offer me treatment if I didn’t go with him and I continued to tell him that even our baby will be affected if he doesn’t come for treatment. So when I told him all that, he got convinced and came.”. "*(Female, in their 30’s, partner returned, accessed ANC at Site 3).

Participants described ‘bad outcomes’ resulting from getting no treatment of syphilis, including the death of a baby and its mother, re-infection, persistent signs and symptoms and medicine may become ineffective.

*“The man can re-infect me since he has not yet been on treatment,” "*(Female, in their 30′, partner did not return, accessed ANC at Site 1).

### Facilitators to returning after notification; perceived benefits of partners’ syphilis treatment

Some men desired to protect the mother and baby during pregnancy and thus came for treatment after notification;

*“She was pregnant and almost giving birth and I knew that it is important for me to come and attend ANC clinic with her. I have to be there for her.”* (Male, in their 30’s, returned, partner accessed ANC at Site 1).

*“My wife had already got treatment for that disease so when she told me, I had to also come and get treatment so that we save our unborn baby”.* (Male, in their 40’s, returned, partner accessed ANC at Site 3.

### Facilitators to returning after notification; reminders by partners and health worker

Male participants who responded to notification mentioned they were reminded by partners, by counselors on phone, and that they had time off work.

“*I first refused, the reason why I refused is that; as you know when you are not feeling sick in any way, you don’t feel like you have to go for treatment but my wife kept on telling me that I also have to go.*” *"*(Female, in their 20’s, did not return, partner accessed ANC at Site 1).

*“I had to come because I was called (by the medical personnel). Even at first, if I had been called, I would have definitely come. I hardly slept last night thinking about coming to hospital so there was no way I would miss coming.”* (Male, in their 20’s.

, did not return, partner accessed ANC at Site 1).

Other men reported that positive health seeking behavior made it easy for them to go to the clinic for care. *“Generally it’s helpful if one cares for their health, like I came and received treatment here and I was told to come back on Saturday.”* (Male, in their 40’s, returned, partner accessed ANC at Site 1).

### Barriers to returning after partner notification; communication, fear of conflict and domestic violence

Many women described experiences of partner notification as a ‘difficult time or ‘hard moment’. Women described they were fearful to notify their partners. Some reported that they overcame the fears, ‘were strengthened’ and notified their partners. Some women chose not to inform their partners at all because of this fear. Some women explained this fear to notify their partners to be because their partners ‘were difficult’. They described their male partners in regard to partner notification using words such as “tough, difficult, and hard hearted”. A women narrated;

“*… he is a very tough man but I later decided to be strong and told him …*” (Male, in their 30’s, returned, partner accessed ANC at Site 1).

A woman who did not notify their partner said;

*“He is a very tough man and wants to do things his way so it is hard for me to talk to him about some things, now like this one; that he is wanted in the ho*spitals... *… my husband is hard to deal with* (difficult) *there was no way I could tell him …*” (Female, in their 30’s, partner did not return, accessed ANC at Site 1).

*“The problem I have is that the man is really hot tempered, so I said if I start on this issue, won’t he throw my stuff outside? I considered that if I start explaining, even the little peace I had would disappear.”* (Female, in their 30’s, partner did not return, accessed ANC at Site 1).

*“I first feared to tell him because he’s a very tough man but I later decided to be strong and told him that he was needed at the hospital to be treated for syphilis. What hurt me a lot was when I found out that he has 4 other women. I really started feeling bad and I know that he has other women minus the 4 that I already know of”.* (Female, in their 30’s, partner did not return, accessed ANC at Site 1).

### Barriers to partner notification; Men’s perceptions of violence and communication skills

Women’s views were in accordance with men’s reports regarding violence and threats of violence at home. Some men mentioned that women in their community fear men because of men’s unpredictable reactions. One man narrated that his wife fears him and that is why she probably did not inform him;

*“Aaaah, the reason I didn’t come was that those times my wife was afraid of me and even when she was pregnant I could hardly get time to come and given her condition, she was afraid of me, not knowing whether to tell me; fearing I might* go out of my mind.” *"*(Female, in their 20’s, partner returned, accessed ANC at Site 3).

Some men suggested that if men were more willing to communicate well with their partners, it would help the woman to disclose and notify them. *“Sometimes, the men are so difficult that their wives fear them; they can’t speak to them nor can they listen to their views. Sometimes it’s also in the way both partners associate with each other. Having mutual understanding is very important for my case.*” (Male, in their 40’s, returned, partner accessed ANC at Site 1).

Several men reported lack of adequate information from their women partners on notification and that the tone of communication from their women partners discouraged some men from coming after notification;

*“The way you see some men in the hospital is not how they behave at home, because you find that they don’t get along with their wives well. So the wives are not free with them; they (women) rudely say," they need you at the hospital without explaining.”* (Male, in their 40’s, returned, partner accessed ANC at Site 1).

*“Some women don’t know how to handle their husbands, you might find some people don’t know how to handle different situations, they end up arguing, pointing fingers to each other that it’s you who brought the disease, how can you start telling me that I am sick? Here it’s because they use a wrong approach.”* (Male, in their 50’s, returned, partner accessed ANC at Site 2).

### Participants’ suggestions for overcoming communication barriers to support notification

Women and men had suggestions for enabling men to come after notification. Women reported that asking health workers to call the partners helped;

*, “I used the health worker, I used a lot of wisdom, I told the counselor to call him and inform him that he is needed. So he called him and he came, it took him a short time to be worked on, good enough I was pregnant and he wanted a child, so whatever I told him to do he accepted.”* (Female, in their 20’s, partner did not return, accessed care at Site 1)*.*

Other women used the hospital documents issued to assist in notification of partners. One man reported;

*“She told me face to face. When she came home from the hospital, she showed me the documents and also informed me that I needed to go to hospital as well and I agreed.” "*(Female, in their 20’s, returned, partner accessed ANC at Site 3).

### Socio-economic factors and access to STI services

Women living separately from partners reported they were unable to notify their partners. Women explained that when partners do not stay under the same roof daily, “it is difficult to communicate to them”.

*“He would never come; he would even switch off the phone given the fact that we don’t stay together.” …*" (Female, in their 30’s, partner did not return, accessed ANC at Site 1).

Many men and women reported that the nature of men’s work precluded their coming to the clinics after notification;

*“The problems of men are many, where you work you are in debts, it may require your physical presence. Like now I have come but I am on tension imagining how many customers have gone”* (Male, in their 50’s, did not return, partner accessed ANC at Site 1).

*“What disturbs most men is time, as you know men we work very hard to cater for our families.”* (IDI*,* male, in their 50’s, did not return, partner accessed ANC at Mulago).

Employed men reported they had to be at work all day said that they did not like to lose money or waste time waiting in clinic queues.

*“Being an employee leaves little or no room to get away from workplace to visit the hospital.”* (Male, in their 50’s, did not return, partner accessed ANC at Site 1).

Many men who owned their own businesses were also unable to leave work as they were the sole persons to attend to customers. Some self-employed men however, felt they were more flexible and gave it as a reason for their coming;

“*… being self-employed, I can make my own schedule and be able to come, someone else may have a boss who wants him to be at work by 8:00am and may not be able to come.”* (Male, in their 30’s, returned, partner accessed ANC at Site 1).

Men who had tested negative for syphilis in other clinics were not keen to seek treatment again with a negative test result;

*“I was hesitant, so I showed her the testing slips from St Mark. So I told her to take the receipts to Mulago for sure I did not come because I did not want to miss work twice, me individually, I had no problem.”* (Male, in their 40’s, did not return, partner accessed ANC at Site 1).

Other men cited health system challenges. A male partner who came to the clinic after notification said, *“The antenatal clinic did not have privacy even the women could see my buttocks …*” *".* (Male, in their 40’s, returned, partner accessed ANC at Site 1)*.*

Men felt that they were treated harshly at one of the clinics where the study was based so they avoided the clinic. Some reported that changes in the infrastructure occurred and they could not locate the clinic. Men described difficulty in access to medical care from hospitals and clinics with long waiting periods and reported perceptions of unwelcoming behavior from some clinic staff. Some men mentioned that operating hours of clinics conflicted with their working hours or that the public health system was impersonal and did not meet their needs;

*“ … it is the hospital we no longer want to come to, because the nurses mishandle you, they bark at you...., it’s as if you committed a crime to fall sick. … .for a big number, me inclusive it is the hospital, we don’t not want to come to”.* (Male, in their 40’s, returned, partner accessed ANC at Site 1).

### Strategies for increased men response to syphilis partner notification

Participants suggested interventions to improve syphilis service delivery and increase men’s return after syphilis partner notification. These included; health education, improved communication skills for the notifying partner especially women, direct notification by the medical personnel rather than by women partners, improving health facilities and improved access to STI care (better men-friendly clinic hours, less painful injections, timely care), better trained medical personnel, and educational and community-based interventions.

*“The only way we can convince them to come is by continuously telling them about the diseases; teach them about the bad effects of not treating it. But me I am very positive that he has syphilis. When I told him that if you don’t go to the hospital the doctors can find you at your work place or any other place that is convenient to you, he kind of liked the idea so I am going to go back and still talk to him. ”.* (Female, in their 30’s, partner did not return, accessed ANC at Site 1).

Some men suggested a scaling up of testing, counseling and educational messages about syphilis testing, and alternatives to the painful syphilis treatment injection. They suggested the benefits of syphilis treatment should be included in the information care package to improve men’s response to partner notification and for better management syphilis, *“Testing should be scaled worldwide and made compulsory for all. Borrow from other programs like polio to eliminate syphilis, teach people how they get syphilis and prevent it and tell them how to avoid acquiring it.” ".* (Male, in their 40’s, returned, partner accessed ANC at Site 1).

*“I knew about the injections and everyone knows that the most painful injections are for syphilis, treatment. Other than PPF, the most painful injections are for syphilis...The men should be told not to fear injection they can receive explanations and come.” ".* (Male, in their 40’s, returned, partner accessed ANC at Site 1).

Men suggested infrastructural changes that appeal to men; *“what I suggest, men should have their own side, so that they are not seen by others. Lounges for both men and women should be put in place. The place was really congested.” (Male, in their 40’s, returned, partner accessed care at Site 1.)*

*“One can tell another that the hospital set up in such and such a place is helpful and handles us well. Even explaining in depth the complications of not receiving treatment.”* (Male, in their 50’s, returned, partner accessed ANC at Site 2).

They suggested increasing access to STD services in the community by increasing numbers of STD clinics;

*“Screening and treatment for syphilis should not be found in one place but also the treatment should be in other health centers that can be accessed by men or even other people wherever they are treatment should not be in only one place”.*(Male, in their 30’s, returned, partner accessed ANC at Site 1).

Women also had suggestions; *“We can put advertisements on radio and TV, also need to reach the local people at the grassroots, it can really help them through the Village Health Team (Community Health Team) leaders who you should equip with knowledge about syphilis since in most cases it is them that usually first approach us in our villages.” "*(Female, in their 30’s, partner returned, accessed ANC at Site 3).

*“I want a syphilis treatment center to be in Kampala for easy accessibility. The cost should be affordable, I hear that the syphilis injection is very costly.” *(Female, in their 20’s, partner returned, accessed services at Site 1).

*“The health workers and the drugs have to be there and health workers should talk to the patients well and not be rude. Then as we are waiting in the queue there should be a television educating us about syphilis and there should be someone updating us about what is going on like someone telling us, “please be patient, they are going to work on you.” That can encourage other people to also come for treatment”.* (Female, in their 20’s, partner returned, accessed ANC at Site 1).

### Men who came to the clinic but did not get treatment

There was a group of men who reported to the clinic but did not get treatment, Some moved away because they realized that they were now in a women’s clinic and did not like it and some said they ran away because of fear of the painful injection. One man said that a staff member wanted to treat him without doing a screening test and he refused.

In summary, our study showed that many factors affected men’s return to the clinic after syphilis partner notification. Using the framework of Axel Roege and colleagues, these factors can be grouped into three major areas; Characteristics of Subject, Characteristics of the Disease, and Characteristics of the Health Services. Under these major categories are the up-growing factors that facilitated and hampered the process of seeking for and receiving the service of treatment for syphilis after Male Partner Notification. (See Table 2). Below these sub-categories were sub-themes of knowledge and perceptions about syphilis, men’s perceptions of the association of syphilis with HIV testing and the stigma associated, fear of painful syphilis treatment, women’s not notifying partners because of fear of their men partner’s reactions to notification, lack of good communication and of communication skills (Individual characteristics). The mode of treatment of syphilis, and men’s understanding of syphilis symptoms and consequences (characteristics of the disease) also affected return. Access to good STD care (access to clinics and compassionate health services) was found to be important as well; the study showed challenges and limits in access for STD care in the health system; some men who were able to return after partner notification still did not receive the STD treatment (characteristics of the health services).

**Table 2:** Summary of reported reasons why men did not attend clinic for syphilis treatment

Summary of Reasons reported why Men did not attend clinic for syphilis treatment	
1.0 Individual Characteristics	
1,1 Knowledge of Syphilis as Disease	
• Ignorance	
• Think women's treatment implied own treatment	
• Men think syphilis is a women’s disease	
1,2 Fear and Stigma	
• Not ready to be associated with HIV.	
• Fear of being arrested, due to unwanted pregnancy /illegal pregnancy	
• Self-medication	
• Fear of self and community stigma, Fear to be seen getting free treatment, would be laughed at	
• Alternative medicines e.g. herbal usage	
1,3 Work/Time Issues	
• Very busy work schedule being an employee leaves little or no room to get away from workplace	
• Non-disclosure at work place to seek permission to go to clinic	
1,4 Communication and Sexual Relationship Issues	
• Was not informed by wife.	
• Men’s ego	
• Blamed women for being promiscuous	
• Women suffered from domestic violence and found difficulty in convincing men	
• Unresolved marital conflicts	
• Poor communication skills by women.	
• Fear partner notification especially in polygamy	
2.0 Characteristics of Disease	
• Disease while asymptomatic, men don’t not see need to seek care /come to clinic	
• Fear painful injection for syphilis treatment, Fear of injection some left clinic in fear	
• Fear of cost of treatment	
3.0 Characteristics of Health Services	
3,1 Perceptions of Health Services	
• Negative attitude towards public facilities ( prefer private clinic)	
3.2 Capacity of Clinics	
• Limited resources of care, crowding, slow services, Left the clinic due to long waiting time, yet had other work to do to earn a living, Was not screened before being asked to receive treatment	
3.3 Accessibility	
• Poor accessibility of the clinic, failed to locate it, Failure to locate clinic	
• Getting a negative syphilis test from elsewhere (validation test).	
• Fear of lack of drugs at facility/No trust in clinic to have needed drugs	

## Discussion

A number of perspectives were shared by both men and women participants regarding the reasons for the low proportion of partners who came to the clinic after syphilis notification in the Syphilis Treatment of Partners (STOP) trial. Reasons cited for not-returning of partners after partner notification by women were; inadequate knowledge about syphilis infection and its treatment, none-notification of partners by the participant women because of fear of violence and fear of conflict with the male partner, limited communication skills with their male partners and lack of proximity to partners (e.g. not living in same household). Other factors influencing male partners’ non-attendance to clinic after notification for syphilis treatment were; busy work schedules, fear of the painful syphilis treatment injection and the associated stigma of syphilis with HIV. These findings are generally similar to a recent systematic review who found fear, stigma and socio-cultural issues were barriers to partner notification of STIs in developing countries [7, 8].

Among partners who reported after notification, knowledge about syphilis as a communicable disease, that treatment was available, desire for own better health, perceptions of male role as a responsible parent with concern for the unborn child’s health and love of their women partners, were facilitators that increased their capacity to make good health choices and come for treatment after notification. Persons who understood syphilis as a transmissible disease were also more likely to come for treatment after notification.

STD services factors like; access to good hospital services, having men friendly clinic hours that were open when needed, professional staff, painless drugs and fast and respectful STD treatment services were reported by men to affect their clinic attendance for syphilis treatment after notification.

In our study, the majority of women interviewed reported experiencing domestic violence. In a study by Decker and colleagues, intimate partner violence (IPV) was a clear barrier to partner notification of STI [9]. Decker’s study suggested that an assessment of intimate partner violence be included in order to minimize harm from partner notification. If a significant risk of IPV is detected, health care workers can provide mechanisms to address related fears concerning partner notification in order to improve effectiveness of STI control and minimize violence to the partner.

Poor communication between partners was reported as a barrier to notification and attendance. Either women were not able to communicate to their partner about the notification, or when communicating they conveyed the message in a way which did not facilitate the men to attend. Some men participants suggested communication training for women and their partners. This finding is backed by the quantitative work of the STOP trial, which showed that only 69 of 81 women whose partner returned were aware that their male partner had attended a clinic for treatment. There is evidence that training in communication skills makes notification easier [8]. Structural health system reasons for not attending the clinic after notification included reports from a few men that they attended the clinic but did not get treatment, or that they were unable to locate the clinics. Similarly, a review found that in developing countries, limited access to the few STD clinics hampered return of notified partners and led to non-treatment [7].

Strategies are needed that address knowledge gaps among men regarding syphilis, that reduce intimate partner violence, target and reduce communication gaps between sexual partners and improve infrastructure and services for STD clinics (including clear directions. Other strategies that increase information on syphilis and sexual health targeted to men with the use of modern technological methods could achieve this efficiently.

Syphilis treatment world over consists of parenteral use of Benzathine penicillin, a painful injection. In cases of allergy to penicillin, other antibiotics have been used but the drug of choice remains Benzathine penicillin. Counseling targeting men’s fear of the painful injections could facilitate return and future drug development of less painful injections for syphilis is crucial as fear of a painful injection was commonly mentioned.

Strategies for improvement of men’s return to clinics for treatment after partner notification that have worked in other settings include strategies involving the partner in “home delivery of partner therapy, home sampling and providing additional information for partners” [10]. These strategies could be explored in Sub-Saharan populations to improve male partner return after notification. A review by Hogben and colleagues showed that expedited partner therapy (EPT) which is the delivery of treatment to a partner and interactive counseling improved notification [11]. although this has been criticized by some. In our study, participants similarly suggested a more active role for health workers in notifying sexual partners (calls and physical visits), suggesting it could reduce the stress associated with informing partners. Similarly, provider based referral interventions have been found to be efficacious in scientific reviews. Programs that involve clinical providers in notification can be instituted or strengthened alongside patient centered notification interventions. Notified partners, are either provided with treatment at home or are taken through an expedited process of treatment at STD clinics rather than wait for long hours in line to improve partner treatment.. In the same study, Hogben and colleagues suggested a role for modern technologies (phones, websites, messaging) in partner notification. These could be instituted in STD programs in Uganda and other developing countries where mobile phones and internet coverage is fairly high and has been found useful in improving participants’ return to clinics for other illnesses [12].

Male partners’ work schedules and work environment need to be addressed; balancing economic losses against the gains of attending clinics. Strategies to get more men to come to STD clinics after notification that take into account patient social, cultural and economic needs are needed. Counseling for women who test positive for syphilis should include improving communication skills to enable easier and more effective partner notification. Client-oriented counseling that takes into account the unique challenges of each infected partner network could be implemented [7]. Table 3 outlines suggested strategies from this study and from the literature to address this. A study in Zimbabwe by Moyo and colleagues showed that partners that received specialized (client oriented) counseling to overcome perceived barriers, [13] had improved STD partner notification. Similarly programs providing STD counseling targeting barriers to notification could be provided to women to improve likelihood to notify male partners.

**Table 3:** Strategies proposed for improving partner notification in male partners of antenatal mothers found syphilis positive in the STOP qualitative study, Kampala, Uganda

	Variables Of Interest	Barriers and Facilitators	Strategies Proposed
Characteristics of Subject			
Age and Gender	Male		Target Programs to Specific Age groups and Genders
	Female		
Socio economic status	• High income • Low Income • Affords Cost of going to Clinic • Cannot Afford cost	**Barriers** Cannot afford cost of transport to clinic, Cannot afford wait at clinic.* High income may have even more stigma to attend government clinics **Facilitators** Can Afford cost of attending, Can afford drugs	Incentivise Return after Notification, Establish Community based clinics Information to |Reduce Stigma Expedited Partner treatment **
Participant’s Knowledge about Syphilis Disease	• Know about Mode of transmission • Hereditary • Sexually Transmitted or Not • Perception of syphilis and HIV/AIDS • Knowledge of signs and symptoms of syphilis • Knowledge of Painful Treatment of Syphilis	**Barriers** Perception that transmission is hereditary* Fear of HIV/AIDs Diagnosis* Stigma* **Facilitators** Factual Knowledge of Transmission* Factual knowledge of signs and symptoms of syphilis** No Stigma	Provision of Information Brochures on Syphilis and its Transmission Increase awareness of Curability of Syphilis Provide Information on STDs and Syphilis Disease Counseling of STD index partners on Syphilis Increase Access to Syphilis Information especially using Modern Technologies; Phones, mails, Websites, Social Media
Individual’s Ability to Leave Work to go to clinic	• Self Employed • Employed by Others • Time off • Working Hours	**Barriers** Employed with long working hours* No weekdays off Clinic closed on Weekends* Cannot afford long wait even if self employed* **Support** Works at night Has time off in week	To provide Expedited Partner Therapy (EPT) where allowed To prioritize Notification Retuness (Not wait in line) To provide open STD clinic hours after work To provide Weekend Open STD Clinics Incentivise Clinc visits after Notification for Men
Perceptions and Practices of Notified partner towards their Partner	• Partner Violence • Good communication with Partner • Value partner • Value Unborn Baby	**Barriers** History of partner violence Fear of partner* Fear of partner violence* **Facilitators** Perceived benefits of syphilis treatment* Good Communication skills** Client Oriented** Counseling Values unborn baby* Values partner*	Community based Programs to Reduce GBV and especially in respect to STD disclosure Provide Client Oriented Counseling to address perceived barriers before notification with index partners Increase knowledge of benefits of Syphilis treatment to Unborn babies and Infected Mothers** Increase Knowledge about benefits of Syphilis Treatment to Expectant mothers**
		Desires Baby and Mother to be healthy* Values Relationship (describes relationship as loving)*	Increase knowledge about benefits of healthy sexual relationships Increase capacity to communicate effectively in STD infected clients Increase Clinician Involvement in Partner Notification Physical
Physical Set up of Life with Partner	• Lives with Partner •Lives separate from partner •Time with Partner (travel. work etc)	**Barriers** Stay separate houses **Facilitators** Good communication skills* Counseling for partner* Trained counselors*	Increase Clinician *Provided Notification for willing clients Improve Contact tracing programs Improve STD surveillance programs with appropriate follow up of all persons in sexual network Provide STD Counseling targeted to separate Living Situations to Clients
Characteristics of the Disease			
Type of symptoms	• Painful/Non painful • Recognizable to partner or others • Disfiguring • Curable /Not curable • Rare/Not rare • Long treatment or Short treatment • Treatment Painful or Not painful • Options for treatment available	**Barriers** Non painful disease, Unrecognizable by individual or partner, Not curable (fatalism), Stigma, fear of painful treatment **Supports** Short painless treatment. Various treatment options eg pills, injections, syrups	Increase the Provision of Information about Syphilis and symptom free STD/Syphilis infection Provide counseling for curable and non-curable STD Increase provision of Painless Syphilis Treatments rather than painful injections of Benzathine Peniccilin** Reduce costs of less painful treatment and increase access to those medications**
Community Knowledge about Disease	• Acceptance/Stigma • High level of knowledge about disease	**Barrier** Stigma, Low knowledge, Fears of community about transmission to other members of the community * Fear of fatality by community **Facilitators** Knowledge of syphilis*	Increase knowledge about Syphilis in Community especially males** Increase acceptance and reduce Stigma of syphilis and STD in community** Increase Capacity of Community clinics to treat syphilis well and efficiently**
Characteristics of the Health Services			
Accessible STD services	• FA	**Barriers** Far from community, easily identified as STD clinic, expensive, opens only on weekdays, staff cannot speak language **Facilitators** Nearby clinic, opens at hours that can meet clients needs, opens on weekends or late evening	Increase Multiple community STD care services availability** Increase opening hours of STD clinics to accommodate men’s working hours*
Capacity of Clinics	• Capable Staff Trained and Knowledgeable on STD Management • STD specific Counseling • Able to treat STD promptly and appropriately Separate Clinics for Men and Women • Availability of STD Medicines	**Barriers** Health workers with limited capability, and training, Limited recent updates in training Laboratory services not adequate, No timely return of laboratory results, poor clinic set-up **Facilitators** Separate clinics for men and women. Confidentiality STD Medicines available	Improve capacity of health care workers providing STD and syphilis care Improve Infrastructure for STD Clinics,; Better privacy and set up Improve laboratory services** Provision of Home based STD sample collection and testing** Improve capacity of health workers to provide efficient male -oriented STD care Improve Surveillance Systems and Contact tracing capacities of clinics and hospitals
Perceptions and Practices of Health Workers	• Trained Staff with Perceptions that reflect training in support for Syphilis Testing and Treatment	**Barriers** Rude staff, Impatient, Poor confidentiality and not attentive*, men treated in antenatal clinic* Health workers not aware of their role in Partner Notification **Facilitators** Trained staff* separate clinics, confidential clinic premises	Train STD staff in provision of efficient ethical services** Provide separate treatment clinic space for men even though partners of Antenatal Women** Increase access to basic STD care and services for all groups men, women and all high risk groups and especially partners of infected persons(both men and women)** Train STD care professionals in provision of surveillance an notification services as well as in contact tracing especially of men** Improve capacity of clinics to provide standardized STD/syphilis care to men and women**
Legal Frameworks and Policies on STD Management and Partner Notification	• Protocols Developed for Surveillance • Policies Developed and In Pace for STD Control and Management of Partner Transmission • Legal Mandate of Medical Personnel to report Notifiable diseases • Systems response for reported diseases as detailed by medical and legal protocols that are ethically optimized • Legal consequences of actionable practices • Maximizing Rights and Health within the policies and frameworks of STD control	**Barriers** No or limited legal frameworks supporting mandatory notification of partners as part of control** No notifiable STD disease component in Surveillance protocols** No training on protocols if exist **Supports** Established Surveillance systems. Legal frameworks and policies eg classification of diseases as notifiable, and mandatory notification of partners within established and humane protocols. Training of personnel on the frameworks and policies. Negative legal consequences for non- notification of known partners	Set up sustainable STD surveillance programs* Enhance Notification Programs and their linkage to other organs of governance** Set up frameworks that capitalize on existing laws to protect the rights and health of persons with STD or syphilis to access care and control STD** Strengthen legal frameworks or their implementation in National and local program for STD prevention and management**

*From Participant data, ** From the literature, From other programs but proposed for the Ugandan context (Based on Kroeger A. Anthropological and socio-medical health care research in developing countries. Social science & medicine 1983;17(3):147-61)

Provision of information through partner -delivered brochures and the use of web based STD information services [14] was found to be acceptable. These too, could be used to improve partner notification in Uganda and other resource constrained settings.

Men who reported to clinics after notification and left without treatment constitute an important group that needs to be targeted. They illustrate system inadequacies in meeting the STD care service needs of men who have overcome other barriers to access. Targeting this gap in access to STD care, in the system efficiencies and in the capacity of clinic personnel to ensure such willing men get timely and good treatment is very important and a reachable goal for improvement. Programs to improve acceptable, timely and efficient access to STD care in clinic and hospital set-ups are crucial.

Globally it is accepted that STD and HIV partner notification is key to epidemiological and public health control of STD and HIV. Some countries have legal frameworks that support partner notification of STD and HIV. In the USA, for example, federal states have the legal authority to notify partners of STD and HIV infection of their spouses. The Ryan White CARE Act Amendments of 1996 that supports "administrative or legislative action to require that a good faith effort be made to notify a spouse of a known HIV-infected patient that such spouse might have been exposed to the human immunodeficiency virus and should seek testing, is an example. The Center for Disease Control (CDC) recommends partner services for all persons newly tested positive for HIV, gonorrhea, chlamydia and syphilis [15].

In many states in the USA, reporting of STD and HIV by physicians is mandatory though a good faith effort is first required of the index partner [16]. These requirements are not agreed upon by many but continue for the public health. Even in such countries where physicians are legally mandated to report STD infection to partners and the public health authorities, reporting is low [17].

In many resource-constrained environments like Uganda, the legal and policy framework for STD partner notification and necessary treatment is very limited. The Uganda HIV AIDS Prevention and Control Act was approved in 2014. The law requires the mandatory notification of HIV status to partners. However this law does not make a mention of STD. Developing countries like Uganda therefore still have barriers to partner notification for STDs and many lack a firm legal and policy framework that addresses the epidemiological control aspects necessary for STI control (testing and treatment of sexual partners). Treatment guidelines in Uganda, mention partner notification and contact tracing but no policies or legal frameworks to mandate treatment and follow up of infected partners exist. Programs for contact tracing are very few and have limited resources. This is a gap in the effective STI control in Uganda and other resource constrained settings. An amendment to the HIV or other communicable disease health acts that addresses STD notification and control is needed.

Our study identified intimate partner violence (IPV) as a factor negatively affecting notification. However, one of the study limitations is that the study did not delve deep enough into the area of IPV. More detailed studies on impact of IPV on STD notification in this setting are needed. Another limitation is that the study did not use more detailed theoretical frameworks in its conceptualization and protocol development. Future studies could employ the COM-B theoretical domains framework [18] to better identify barriers and facilitators of uptake of STD notification services. However the study was able to highlight significant issue in the area of STD notification in male partners and provides important data to inform public health STD control in Uganda.

## Conclusions

In this study we found that the notification of male partners by pregnant women treated for syphilis was low due to individual characteristics (lack of adequate knowledge about syphilis as a disease, its consequences and treatment in men, fears of domestic violence, and lack of communication between partners; characteristics of the disease (signs and symptoms, type of treatment, effects). Combined with this were structural barriers (characteristics of the health services) that hindered male partner treatment. These factors threaten efforts to eliminate mother-to-child re-infection of syphilis. Improved public messaging about syphilis, especially to men, provision of communication skills to women and men regarding sexual health, improvement of access to quality STD services to men, as well as legal and policy frameworks that can support STD notification and treatment in resource-constrained settings are needed for effective STD control. Further studies will give rise to more effective strategies in the control of syphilis especially among pregnant women, and their male partners.

## Abbreviations

ANC: ante-natal care
IDI: Infectious Diseases Institute
MOH: Ministry of Health
PN: Partner notification
POCT: point-of-care tests
STI: sexually transmitted infections
